# Blood Flow Restriction in Athletic Populations—Part 1: Safety Considerations, and Methodological Frameworks

**DOI:** 10.3390/jfmk11020175

**Published:** 2026-04-27

**Authors:** Chris Gaviglio, Christian J. Cook, Stephen P. Bird

**Affiliations:** 1 School of Health, Psychological and Medical Sciences, University of Southern Queensland, Ipswich 4305, Australia; stephen.bird@unisq.edu.au; 2 Institute for Health, University of Southern Queensland, Ipswich 4305, Australia; 3 Biomedical Discipline, School of Science and Technology, University of New England, Armidale 2350, Australia

**Keywords:** blood flow restriction, KAATSU, safety, arterial occlusion, athletes

## Abstract

**Background**: Blood flow restriction (BFR) training induces morphological and neuromuscular adaptations using low-intensity exercise (20–40% 1RM), offering a reduced mechanical load alternative to traditional high-load resistance training. Safe and effective implementation, however, requires a clear understanding of physiological mechanisms, contraindications, and pressure determination methodologies. In this three-part series, we provide a comprehensive review of BFR for athletic populations and provide strength and conditioning coaches with a structured framework for screening, safety, and methodological considerations to support BFR integration in high-performance settings. **Methods**: A narrative review of the literature examining BFR safety, contraindication screening, adverse event reporting, and occlusion pressure determination was conducted using a PubMed and MEDLINE search. Search terms included combinations of (“blood flow restriction” OR “BFR” OR “occlusion training” OR “KAATSU”) AND (“safety” OR “contraindications” OR “risk stratification”) AND (“arterial occlusion pressure” OR “limb occlusion pressure” OR “occlusion pressure” OR “Doppler” OR “handheld Doppler” OR “pulse oximetry” OR “cuff width” OR “capillary refill time” OR “monitoring”). Studies examining contraindication screening systems, arterial occlusion pressure calculation methods, and real-time monitoring protocols were evaluated. Primary considerations included risk stratification frameworks, pressure determination accuracy, and control parameter validation for ensuring vascular safety during application. **Results**: Risk stratification systems can effectively identify absolute and relative contraindications requiring medical clearance prior to BFR use. Epidemiological data indicate that adverse events are transient and non-serious, while serious events appear rare when evidence-informed protocols are applied. Doppler-based assessment remains a criterion approach for determining inflation pressure, although validated estimation methods using limb circumference and systolic blood pressure offer a pragmatic and comparable alternative for applied environments. Inflation pressures of 50–80% arterial occlusion, adjusted for cuff width, produce effective and safe stimulus. Real-time monitoring through capillary refill time, pulse strength palpation, and skin coloration can support iterative pressure optimization and help identify excessive restriction pressures. **Conclusions**: BFR implementation in athletic populations requires systematic screening protocols, individualized inflation pressure determination using validated methods, and real-time monitoring parameters. These foundations provide the essential safety infrastructure required before progressing to specific training applications across resistance, cardiovascular, and other performance and rehabilitation modalities.

## 1. Introduction

**Figure 1:**
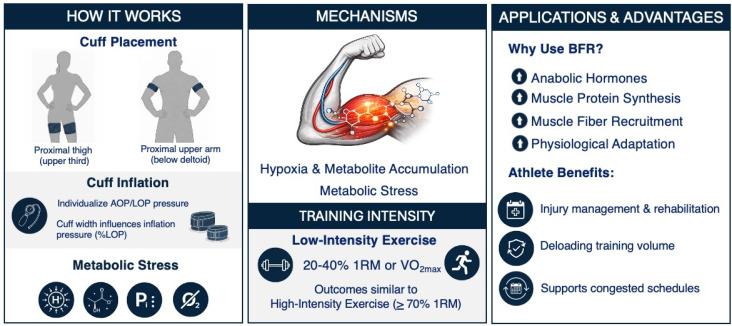
Conceptual overview of blood flow restriction training mechanisms and applied use in athletes. Abbreviations: AOP, arterial occlusion pressure; LOP, limb occlusion pressure; RM, repetition maximum; BFR, blood flow restriction.

Blood flow restriction (BFR) training is a specialized training method where external pressure is applied to the most proximal region of the arms or legs to partially restrict arterial inflow and restrict the venous outflow in working musculature during exercise [1]. This restriction is produced using a specialized cuff or belt, commonly via a pneumatic tourniquet system [2]. Originating in Japan in the 1960s and known as KAATSU training, its development preceded modern BFR research and established principles that continue to influence contemporary practice of strength and conditioning coaches (S&C) today. When the cuff is inflated, gradual mechanical compression of the underlying vasculature allows arterial blood flow to continue to reach the distal tissues while limiting venous return. Compression of the vasculature proximal to the skeletal muscle results in a subsequent reduction in oxygen availability to create a local hypoxic environment within the muscle [3,4]. This, in combination with the accumulation of metabolic by-products (e.g., inorganic phosphate, hydrogen ions, and lactate) [5,6], generates a potent metabolic stress that contributes to downstream adaptive responses. Additional proposed mechanisms underlying BFR-induced adaptations include increased anabolic hormone concentrations, intramuscular signaling (independent of hormones and growth factors), enhanced muscle protein synthesis, reduced muscle protein breakdown, upregulation of heat shock proteins, increase satellite cell proliferation and activity, and muscle fiber recruitment [6,7,8]. Collectively, these mechanisms provide the physiological basis for the hypertrophic and strength responses observed in BFR training (Figure 1). For detailed mechanistic summaries, see Loenneke et al. [7] and Pearson and Hussain [9].

A key advantage reported for BFR training is its capacity to elicit significant muscular adaptations when combined with low-intensity exercise, typically resistance exercise performed at 20–40% of one-repetition maximum (1RM) or cardiovascular exercise performance at 30–50% of maximal oxygen consumption (VO2max) [10,11,12,13]. This contrasts traditional high-load resistance training, which requires loads ≥ 70% 1RM to stimulate similar increases in muscle size and strength [14]. The reduced mechanical loading associated with BFR training is particularly valuable for athletics populations, providing a means to decrease joint stress, minimize muscle damage, and limit systemic fatigue, while still promoting meaningful adaptation [15,16]. This enables coaches to maintain and/or progress the training stimulus during injury, rehabilitation, deloading periods, or phases of congested competition [17]. As BFR can be applied across resistance, aerobic and anaerobic exercise modalities, as well as in passive settings, it represents a versatile tool for managing athlete load while supporting those seeking to optimize training adaptations while managing athlete load [15,16]. Collectively, this highlights BFR as an effective adjunct to conventional training methods, especially when high mechanical loads are impractical or contraindicated [16,17].

Within athletic populations, high-performance environments, and rehabilitation settings, the appeal of BFR lies not only in its capacity to produce meaningful adaptations at reduced mechanical loads, but also in its ability to strategically manage training stress across varied phases of a season. However, the physiological mechanisms that make BFR effective also necessitate a clear understanding of safety parameters, screening processes, and appropriate pressure prescription. These foundational elements are critical to ensure that BFR implementation is both effective and aligned with athlete health and performance. The aim of this three-part series is to present evidence-based guidelines and practical application considerations for BFR training, with a specific focus on (1) contraindications and safety considerations required for safe and effective implementation, (2) the application of BFR within resistance training across the full loading spectrum, and (3) the use of BFR for cardiovascular conditioning, performance priming, and rehabilitation. Collectively, this will provide a BFR implementation framework to assist coaches and practitioners with the integration of BFR into high-performance environments while prioritizing athlete health and performance.

## 2. Methods

### 2.1. Search Strategy

A narrative review of the literature relevant to the safe implementation of BFR training was conducted. A comprehensive literature search of PubMed and MEDLINE was performed using combinations of the following terms: (“blood flow restriction” OR “BFR” OR “occlusion training” OR “KAATSU”) AND (“safety” OR “contraindications” OR “risk stratification”) AND (“arterial occlusion pressure” OR “limb occlusion pressure” OR “occlusion pressure” OR “Doppler” OR “handheld Doppler” OR “pulse oximetry” OR “cuff width” OR “capillary refill time” OR “monitoring”). Reference lists of key consensus statements, epidemiological surveys, and methodological validation studies were also screened to identify additional relevant sources.

### 2.2. Study Selection

Studies and source documents were included if they met one or more of the following criteria:

- Addressed BFR pre-participation screening, contraindications, or risk stratification frameworks;
- reported adverse events or safety outcomes associated with BFR or KAATSU training, including epidemiological surveys and surveillance studies;
- Evaluated methods for determining limb or arterial occlusion pressure (LOP or AOP), including Doppler-based methods, handheld Doppler, pulse oximetry, or validated estimation approaches;
- Described monitoring or control parameters relevant to applied BFR use (for example capillary refill time, pulse quality, skin color, or symptom monitoring).

Exclusion criteria:

- Animal-only studies;
- Studies not available in English;
- Studies lacking sufficient methodological detail to inform applied interpretation;
- Reports not related to BFR or KAATSU methodology.

Although the manuscript focuses on implementation in athletic settings, evidence from broader adult populations was retained when it directly informed safety screening, adverse event risk, or pressure prescription methodology. A single reviewer (primary author) conducted the search and study selection, with consultation among all authors for ambiguous cases. We acknowledge that this selective approach may introduce bias toward positive findings, well-established research groups, and may favor studies with positive or applied outcomes.

### 2.3. Data Extraction and Narrative Synthesis

For included sources, we extracted information relevant to:

- Screening and contraindication frameworks
- Safety outcomes and adverse event reporting
- Inflation pressure determination methodology and validity metrics
- Monitoring parameters used to refine and confirm tolerable restriction.

Findings were synthesized narratively and organized by domain (screening and contraindications, safety and adverse events, control parameters, and pressure determination). No pooled statistical analysis was undertaken.

### 2.4. Consideration of Evidence Strength and Limitations

While this review is narrative in design, the search strategy was structured to capture the literature most relevant to safe BFR implementation in applied sport and high-performance environments. A scoping assessment indicated that the evidence base most directly informing coach-facing decision-making is concentrated within three domains: contraindication and risk stratification frameworks, epidemiological surveillance of adverse events, and methodological validation studies for determining limb or arterial occlusion pressure. Our database search, combined with manual reference list screening of key position statements and foundational studies, captured the majority of sources addressing these applied safety and methodological themes. While we did not apply formal systematic review methodology (e.g., PRISMA reporting, multiple independent reviewers, or risk-of-bias tools), the focused nature of the applied safety and methodology literature in this domain allowed this narrative approach to provide thorough coverage of evidence most pertinent to practitioners working with athletic populations, while acknowledging domain-level limitations that may influence interpretation.

## 3. Contraindications to BFR Use

Effective BFR implementation requires a comprehensive pre-participation screening process to ensure the safety of the individual and mitigate potential adverse effects. Extending general pre-participation screening such as lifestyle factors, family medical history, and underlying clinical conditions that may contraindicate BFR use [18], Nakajima and colleagues [19] propose a ‘point-based risk stratification system’ in which a cumulative score of five or more denotes ‘high-risk’ and exclusion from BFR training. This high-risk category includes conditions such as deep-vein thrombosis, hereditary thrombotic tendency; and antiphospholipid antibody syndrome pregnancy, particularly during the third trimester (due to altered coagulation profiles)—considered a relative contraindication; therefore, in principle, BFR training is not advised during pregnancy. Although current evidence does not suggest that BFR exacerbates varicose veins, practitioners should exercise caution and seek medical clearance. Kacin et al. [18] expanded upon this framework by distinguishing between ‘absolute’ and ‘relative’ contraindications, recommending medical consultation when relative risks are identified. Importantly, this points-based approach should be interpreted as a practitioner-oriented risk stratification aid designed to support screening and referral decisions, rather than as a universally accepted clinical guideline. To our knowledge, it has not been prospectively validated against clinical outcomes in athletic cohorts, and it should therefore be used as an adjunct to standard exercise pre-participation screening and clinical judgment. Table 1 summarizes clinical conditions and their associated risk scores. We recommend that coaches use this as a practical guide to determine eligibility and, where indicated, obtain medical clearance.

**Figure 2:**
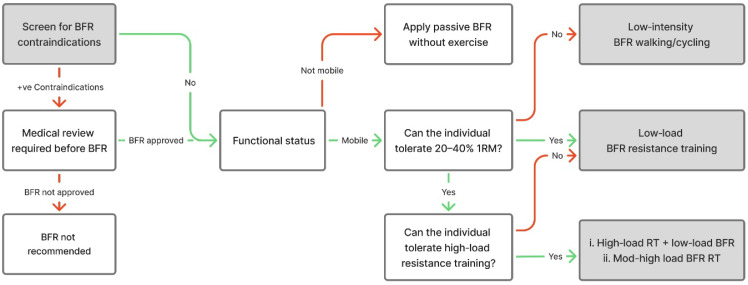
Decision-making flowchart illustrates practical steps for implementing BFR training strategies in healthy and athletic populations. Adapted from Scott et al. [1] and informed by KAATSU implementation principles described by Nakajima et al. [19]. Abbreviations: BFR, blood flow restriction; 1RM 1-repetition maximum; RT, resistance training.

To complement this risk-screening process, coaches also require a practical framework for translating clinical risk information into practical programming decisions. The framework presented in Figure 2 offers a structured decision-making pathway to support coaches in selecting the most appropriate BFR strategy based on an individual’s functional capacity, mobility status, and tolerance to loading. Figure 2 is a practitioner-oriented framework adapted from Scott et al. [1] and informed by KAASTU implementation principles described by Nakajima et al. [19]. It is presented as applied guidance following screening rather than as a prospectively validated clinical decision model. When integrated with the contraindication screening described above, this framework provides a practical means of translating clinical risk stratification into safe and context-appropriate exercise prescription. Collectively, this reinforces the importance of aligning BFR application with individual risk profiles, thereby ensuring an appropriate and safe approach to practice.

### Contraindication Key Points

The following key points outline essential contraindication and risk-screening considerations to guide the safe and appropriate application of BFR training:

1. Prior to implementation of BFR training, complete the risk-screening process outlined in Table 1 to determine medical history, conditions, or diseases that may constitute contraindications to BFR.
2. If the risk magnitude is absolute and/or the participant scores five points or more, BFR should not be applied.
3. If the risk magnitude is relative and/or the user scores four points, consultation with a physician and formal medical clearance required.
4. Once participant receives physician clearance, apply the BFR decision-making flow chart for training intensity and modality (Figure 2).

## 4. Safety Considerations

When prescribed under controlled conditions by trained personnel, BFR training is considered a safe modality for most individuals, regardless of age or training status [2,20]. An epidemiological review of 25,813 individuals reported 1672 instances of adverse events (6.47%), of which 99.4% required no pharmacologic, surgical, or radiographic intervention [20,21]. The most commonly reported side effects were transient subcutaneous hemorrhage (13%) and numbness, affecting between 1.3% and 19% of users [20,21]. In a separate national survey of 12,642 individuals undertaking BFR training, serious complications included venous thrombosis (0.055%), pulmonary embolism (0.008%), and rhabdomyolysis (0.008%), with no long-term complications reported [21]. These findings should be interpreted with the caveat that much of the available safety evidence derives from observational surveys across heterogeneous settings; variation in device type, applied inflation pressure, protocol characteristics, and practitioner training may therefore limit generalizability.

A mechanistic understanding of these risks can further guide risk stratification and safe practice. Intermittent external compression alters local hemodynamics and shear stress, which may be relevant in individuals with thrombotic risk factors; however, serious vascular events remain rare provided that established screening procedures and contraindications are applied during pre-participation screening [4]. Accordingly, practitioners should adhere to recommended continuous restriction duration, avoid pressures exceeding individualized arterial occlusion thresholds, and monitor symptoms of excessive pressure such as pallor, numbness, or dizziness.

In applied sport contexts, additional modifiers warrant attention. Sex-related differences in limb anthropometrics can influence cuff fit and the pressure required to achieve equivalent relative occlusion. Elite athletes sustaining high training stress loads may present with altered autonomic tone and reduced vascular compliance, and environmental stressors, particularly heat and dehydration can amplify cardiovascular strain [4]. Practically, these considerations support adequate pre-session hydration, heighted caution in thermally demanding environments, avoidance of prolonged continuous cuff restriction, and consistent symptom-based monitoring throughout training.

Safety concerns may arise from the proliferation of unregulated BFR equipment and inconsistent application protocols [22]. Integrating a structured BFR pre-screening process, supported by safety principles derived from KAATSU methodology [22], strengthens the foundation for safe and effective BFR implementation.

When using BFR, athletes may find that inflating BFR cuffs directly to their calculated working pressure uncomfortable, particularly if it exceeds may be beyond their tolerable compression limit. This is most evident in novice users or those not adapted to higher levels of limb compression. KAATSU methodology [22] addresses this through a brief rhythmic compression–decompression warm-up cycle designed to prepare the vascular system prior to full inflation pressure. Exercise is not required during this cycle, as the compression–decompression stimulus alone elicits a meaningful physiological response [23]. Although not standard practice in BFR, this graduated pre-inflation strategy offers a practical means of enhancing comfort, improving tolerance, and facilitating a smoother transition to the training inflation pressure.

### BFR Practical Safety Guidelines

The following safety guidelines are adapted from KAATSU methodology and are consistent with BFR implementation recommendations [4,22]. They outline essential considerations to minimize risk and support the safe application of BFR training:

1. Ensuring adequate hydration prior to application.
2. Avoiding cuff placement directly on the skin.
3. Encouraging controlled and continuous breathing during exercise.
4. Avoiding full arterial occlusion or excessive pressures.
5. Limiting continuous use to 15 min for upper limbs and 20 min for lower limbs in one continuous inflation bout.
6. Never applying inflated cuffs simultaneously to both upper and lower limbs.

## 5. Control Parameters

The control strategies outlined in Table 2 provide valuable practical insights for coaches and practitioners wishing to refine pressure selection and compensate for inaccuracies in a single diagnostic method [22]. A fundamental KAATSU indicator is skin color distal to the cuff. When pressure is appropriate, the skin adopts a dark reddish–fleshy color, suggesting partial arterial inflow with venous pooling. Pressures that are too high produce a bluish-purple appearance, reflecting substantial reductions in both arterial inflow and venous outflow, whereas excessively high pressures can result in pallor, indicating near-complete occlusion of the local vasculature. The second method employs capillary refill time (CRT) as a simple assessment of peripheral perfusion. For upper-body training, the CRT is evaluated at the palm of the hand, whereas for lower-limb training CRT is assessed on the quadriceps above the knee or back of the foot/sole. To evaluate CRT, the practitioner applies fingertip pressure to the skin to induce temporary pallor, then observes the time required for the original color to return. Under normal conditions, this occurs within 2–3 s, whereas slower refill time indicates that cuff pressure may be excessively high and restricting local blood circulation. A third criterion is pulse strength, reflecting the pulsatile nature of arterial flow. As cuff pressure increases, distal pulsation becomes stronger until reaching an optimal level. If inflation pressure is too high, the pulsation sensation weakens or disappears entirely, signaling excessive vascular restriction. Finally, as a functional indicator of appropriate pressure, muscular fatigue across each set may be employed. With the correct inflation pressure, repetitions should decline across sets due to the fatigue associated with the increased metabolic stress. If repetitions do not decrease, the inflation pressure may be insufficient to elicit the desired physiological stimulus.

In KAATSU practice, athletes and coaches are also encouraged to monitor subjective stress reactions, including dizziness, nausea, chest discomfort, shortness of breath, or exaggerated fatigue [22]. If any of these arise, BFR should be paused, pressure reassessed, and the session discontinued if symptoms persist. A further response sometimes seen during early use is petechial hemorrhage, caused by capillary rupture beneath the skin. These microbleeds are harmless, typically resolve within days, and do not constitute a reason to cease BFR training [19]. Table 3 provides a summary of safe BFR implementation considerations, including pre-screening, equipment selections, monitoring during use and session safety recommendations.

## 6. BFR Methodology

### 6.1. Determining the Inflation Pressure to Be Used for BFR Training

There remains limited consensus regarding the prescription of a definitive inflation pressure [24], despite the well-established benefits of BFR training [1,15,16]. The first step in determining appropriate BFR pressure is to identify the minimum pressure required to occlude arterial blood flow distal to the cuff, termed arterial occlusion pressure (AOP) or limb occlusion pressure (LOP). AOP can be determined manually or automatically through gradual cuff inflation until pulse cessation is detected using diagnostic equipment such as a Doppler flowmeter or pulse oximeter [25,26,27]. Alternatively, LOP can be estimated using formula-based approaches incorporating the individual’s limb circumference and blood pressure [28]. Once AOP or LOP has been established, training inflation pressures set at 50–80% of the occlusion threshold are widely recommended as safe and effective [1]. This occlusion-anchored, percentage-based approach is specifically intended to account for inter-individual differences in limb size and composition, cuff characteristics, and vascular properties, which is why fixed absolute inflation pressures are generally discouraged. In practice, selection within this range is guided by individual tolerance and training objective, with more conservative pressures typically used during initial familiarization or when screening indicates relative risk factors, and higher pressures used when well tolerated and the goal is to increase metabolic stress at low external loads. Accordingly, accurate quantification or estimation of AOP/LOP is a critical step for individualizing BFR application.

Pulse wave Doppler ultrasound (DU) is viewed as the gold standard for identifying AOP because it enables the direct assessment of arterial flow characteristics including velocity and volume [26]. However, cost and restricted portability restrict its practical use. Handheld Doppler (HH-DU) devices may provide an affordable and accessible alternative. HH-DU provides auscultatory confirmation of pulse presence or absence, without quantifying flow. Despite this, HH-DU demonstrates strong agreement with laboratory-based DU (r ~ 0.94), with minimal mean differences (<6 mmHg) across both upper and lower limbs [26,29]. Intra-rater reliability is also moderate to excellent (ICC = 0.72–0.79), with a between-session variation of 5–6 mmHg [30]. Nevertheless, both Doppler methods require appropriate operator skill to achieve accurate and consistent readings. Accordingly, practical utility across inflation pressure determination methods varies by limb and cuff characteristics, and is influenced by operator skill, which supports percentage-based prescription anchored to an occlusion reference point and refinement via control parameters to minimize the likelihood of excessive restriction.

Pulse oximetry (PO) has been explored as an additional method for determining AOP [25,31]. PO detects changes in peripheral blood volume, with complete occlusion indicated by disappearance of pulsatile waveforms during cuff inflation. In a study of 94 participants, PO produced close estimates of AOP for the upper limbs (mean difference: 1.5 ± 13.9 mmHg; *p* = 0.308), although wider limits of agreement were noted. In contrast, PO overestimates AOP in the lower limbs (mean difference = 8.5 ± 21.1 mmHg; *p* = 0.001), with broad limits of agreement (−33 to ±49.9 mmHg) [25]. These findings support PO as a practical and valid alternative for upper-limb assessments but indicate insufficient accuracy for the lower-limb application.

Circumference- and blood pressure-based calculations offer another viable approach for estimating LOP and are validated in both surgical [27] and BFR training [24,32] contexts. In surgical settings, Tuncali et al. [27] proposed an estimation formula, LOP = (SBP + 10)/K_TP_, where SBP is the patient’s systolic blood pressure and K_TP_ represents a tissue padding coefficient derived from the patient’s limb circumference. This method closely matched Doppler-derived AOP values and reliably produced bloodless surgical fields during upper- and lower-limb surgery, while significantly reducing setup time and technical demands compared with traditional DU methods [27,33]. A subsequent clinical trial in knee arthroplasty further confirmed its validity and efficacy, where the time required to calculate and apply tourniquet pressure was significantly less than for the AOP estimation method (23.9 ± 4.8 s vs. 178.8 ± 25.5 s respectively), while achieving similar arterial occlusion levels and surgical field quality [33].

In BFR-context, Loenneke et al. [24] supported this concept through a large-scale investigation examining the predictors of arterial occlusion pressure across 171 participants. Regression modeling revealed that, for the upper limb, arm circumference (measured at a distance 50% distal to the acromion) and brachial SBP were the strongest predictors of AOP. For the lower limb, thigh circumference (measured at 33% of the distance from the inguinal crease to the top of the patella) was the dominant predictor of AOP [24]. These findings align with the surgical data and reinforce the physiological rationale that both limb size and systemic blood pressure can estimate the minimal occlusion threshold. Collectively, such evidence supports the use of circumference-based estimation methods as practical, time efficient and individualized means of establishing BFR inflation pressures. Once AOP has been determined, applying a percentage of this value, typically 50–80% in consideration of the cuff width provides an appropriate inflation pressure [1]. Wider cuffs achieve arterial occlusion at lower pressures because the force distribution is over a larger surface area [32,34,35]. Although wider cuffs may reduce required inflation pressures, they may restrict movement during exercise, and therefore cuff selection should balance comfort and mobility according to the intended training task. Overall, limb circumference methods, in conjunction with blood pressure assessment, offer a rapid, individualized, and safe means of determining BFR training pressure. Table 4 presents a practical framework for establishing BFR inflation pressure.

### 6.2. BFR Training Pressure Key Points

The following key points summarize the current recommendations for determining BFR training pressures and establishing safe and effective limb occlusion levels:

1. Determining AOP is established through the following:
  (a) Doppler ultrasound (DU/HH-DU): Requires operator skill and access to specialized equipment.
  (b) Pulse oximetry: Practical and valid for upper limbs only, but less accurate for lower limbs.
  (c) Circumference-based estimation of LOP: Rapid, low-cost and validated method for both upper and lower limbs.
2. Recommended Training Pressure:
  (a) Training at 50–80% of calculated AOP/LOP, adjusted for cuff width.

## 7. Conclusions

Blood flow restriction training provides coaches and practitioners with a versatile and effective method for eliciting meaningful muscular adaptations while reducing mechanical loading, making it particularly advantageous during rehabilitation, injury management, and congested competition schedules [17]. However, its safe and effective integration into athletic environments depends on strict adherence to evidence-based screening procedures, accurate determination of cuff inflation pressures, and systematic monitoring of control parameters. This paper has outlined the essential safety framework and methodological considerations that must underpin BFR implementation in athletic environments. Key considerations include thorough BFR pre-participation screening to identify contraindications, the use of validated techniques to determining appropriate cuff inflation pressure, and systematic monitoring of control parameters such as capillary refill time, pulse strength, and distal skin coloration [19,22]. These measures are fundamental to mitigating risk, particularly considering the widespread availability of devices and variable practitioner experience [22,36]. Establishing these foundational principles ensures that coaches can apply BFR with confidence and precision. Parts 2 and 3 of this series will build on this framework by examining the practical application of BFR across resistance exercise, cardiovascular modalities, sport-specific training, and therapeutic rehabilitation contexts, including bone-related and pain-management interventions. Collectively, these papers aim to deliver a comprehensive, evidence-based guide for the safe and effective integration of BFR within athletic performance environments.

## Figures and Tables

**Table 1:** BFR pre-screening: Points-based risk stratification system.

Risk	Medical History of Lifestyle Factor	Points	Notes
Absolute	History of deep-vein thrombosis Hereditary thrombotic tendency Antiphospholipid antibody syndrome Level 1 hypertension (SBP > 140 mmHg)	5	Avoid BFR usage.
	Pregnant women	4	
Relative	Varicose veins of legs Prolonged immobility (incapable of 8 h thromboprophylaxis rehabilitation) Atrial fibrillation or heart failure	3	Seek medical advice according to additive score.
	People aged over 60 years old BMI > 30 Hyperlipidemia, Malignancy Quadriplegia High hemoglobin level	2	
	People aged 40 to 58 years old Women BMI 25–30	1	
Precautionary	History of injury to arteries or veins History to nerves (including back or neck injury) Metal implants Undiagnosed groin/calf pain History of compartment syndrome Recent surgery in last 7 days Hypertension (SBP 120–140 mmHg)		Seek medical advice.

Abbreviations: BFR, blood flow restriction; BMI, body mass index; SBP, systolic blood pressure. Adapted from Kacin et al. [18] and Nakajima et al. [19]. This scoring system is presented as a practitioner-oriented risk stratification aid to guide screening and referral, not as a validated clinical diagnostic tool.

**Table 2:** Key control parameters to refine pressure selection.

1. Skin Color	2. Capillary Refill Time	3. Muscular Fatigue Across Exercise
Optimal inflation pressure = dark reddish–fleshy. Too high = bluish-purple. Excessively high = pale.	Upper body = palm of hand. Lower body = above knee or back of the foot/sole. Optimal CRT = 2–3 s. CRT > 3 s suggests excessive pressure.	Repetitions should decrease set-to-set. Consistent repetition count set-to-set may indicate pressure is too low.

Abbreviations: CRT, capillary refill time; s, seconds.

**Table 3:** Safety guidelines for BFR implementation.

Variable	Guidelines	Comments
Pre-screening	Conduct a medical and training-history screen using absolute/relative contraindications.	Exclude ≥5 points; seek medical clearance at ≥4 points.
Equipment Selection	Use regulated pneumatic systems capable of precise pressure control.	Avoid improvised or uncalibrated devices.
Monitoring During Use	Observe skin color, capillary refill time (2–3 s), pulse strength, and repetition decline.	Deviations indicate under- or over-pressure.
Session Safety	Discontinue if dizziness, nausea, chest discomfort, severe fatigue, or abnormal BP responses occur.	Reassess pressure; stop session if symptoms persist.
Novice User Adjustments	Use a KAATSU-style pre-inflation cycle before full working pressures.	Enhances comfort and tolerance, especially for novice users.

Abbreviations: s, seconds; BP, blood pressure.

**Table 4:** Framework for establishing BFR inflation pressure.

Step	Procedure	Comments
Step 1: Measure BP	Measure brachial SBP and DBP in a seated position, arm supported.	Seated BP aligns with the posture used in most resistance or aerobic BFR tasks.
Step 2: Measure limb circumference	Arm: 50% of acromion–olecranon distance. Thigh: 33% of distance from inguinal crease to top of patella.	Tape measure should be snug but not compressing soft tissue.
Step 3A: LOP—Loenneke [24] equation	Upper Limb: 0.514 × SBP + 0.339 × DBP + 1.461 × Arm Circ. + 17.236 Lower Limb: 5.893 × Thigh Circ. + 0.734 × DBP + 0.912 × SBP − 220.046	Strong predictive accuracy in resistance-trained and recreational cohorts.
Step 3B: LOP—Tuncali [28] method (alternative)	LOP = (SBP + 10)/K_TP_ K_TP_ based on circumference ranges	See Tuncali for K_TP_ values
Step 4: Determine inflation pressure (%LOP)	Select %LOP based on cuff width and training modality: Wide cuffs (10–13.5+ cm): 40–60% Medium cuffs (7–9 cm): 50–70% Narrow cuffs (≤5 cm): 70–80%	Cuff width modifies the required %LOP: wider cuffs require lower pressures. Applies to resistance, aerobic and IPC protocols *.
Step 5: Adjust using control parameters	Evaluate the following: Skin color: reddish–fleshy = appropriate; bluish/purple = too high; pale = excessive occlusion. CRT: 2–3 s normal; >3 s = excessive pressure. Pulse strength: strongest pulsation = suitable; absent = excessive occlusion. Repetition decline: reps should drop set-to-set; constant output suggests insufficient pressure.	These controls compensate for any slight estimation error and refine individualization.
Step 6: Optional pre-inflation cycle	Use rhythmic inflation–deflation cycles before reaching full pressure.	Beneficial for new users or athletes sensitive to higher pressures. Improves tolerance and comfort.

Abbreviations: BP, blood pressure; SBP, systolic blood pressure; DBP, diastolic blood pressure; Circ, circumference; LOP, limb occlusion pressure; K_TP_, tissue padding coefficient; s, seconds; *, IPC protocols typically require higher %LOP.

## Data Availability

No new data were created or analyzed in this study. Data sharing is not applicable to this article.
